# Supplementary material for: A Machine Learning Model for Predicting Hospitalization in Patients with Respiratory Symptoms during the COVID-19 Pandemic
Source: J Clin Med. 2022 Aug 5;11(15):4574. doi: 10.3390/jcm11154574 (PMC9369854; doi:10.3390/jcm11154574)
Supplement: Supplementary file 1 [file jcm-11-04574-s001.zip › jcm-1829514-supplementary.pdf]

A Machine Learning Model for Predicting Hospitalization in Patients with Respiratory Symptoms during the COVID-19 Pandemic

Victor Muniz De Freitas [1], Daniela Mendes Chiloff [1], Giulia Gabriella Bosso [1], Janaina Oliveira Pires Teixeira [1], Isabele Cristina de Godói Hernandes [1], Maira do Patrocínio Padilha [1], Giovanna Corrêa Moura [1], Luis Gustavo Modelli De Andrade [2], Frederico Mancuso [3], Francisco Estivallet Finamor [3], Aluísio Marçal de Barros Serodio [4], Jaquelina Sonoe Ota Arakaki [5], Marair Gracio Ferreira Sartori [6], Paulo Roberto Abrão Ferreira [7] and Érika Bevilaqua Rangel [8,*]

[1] Paulista School of Medicine, Hospital São Paulo, Federal University of São Paulo, São Paulo 04038-901, Brazil
[2] Department of Internal Medicine, Botucatu Medical School, University of São Paulo State, Botucatu 18618-687, Brazil
[3] Discipline of Emergency Medicine, Department of Medicine, Paulista School of Medicine, Hospital São Paulo, Federal University of São Paulo, São Paulo 04038-901, Brazil
[4] Sector of Bioethics, Department of Surgery, Paulista School of Medicine, Hospital São Paulo, Federal University of São Paulo, São Paulo 04038-901, Brazil
[5] Pneumology Division, Department of Medicine, Paulista School of Medicine, Hospital São Paulo, Federal University of São Paulo, São Paulo 04038-901, Brazil
[6] Department of Obstetrics, Paulista School of Medicine, Hospital São Paulo, Federal University of São Paulo, São Paulo 04038-901, Brazil
[7] Infectious Disease Division, Department of Medicine, Paulista School of Medicine, Hospital São Paulo, Federal University of São Paulo, São Paulo 04038-901, Brazil
[8] Nephrology Division, Department of Medicine, Paulista School of Medicine, Hospital São Paulo, Federal University of São Paulo, São Paulo 04038-901, Brazil
* Correspondence: erikabr@uol.com.br.

Table S1. Comparison of demography, signs, symptoms, and comorbidities between patients who required hospitalization and those who did not require hospitalization from March to August-2000 at Hospital São Paulo, Brazil.

Variables	No Hospitalization (n=6596)		Hospitalization (n=740)		*P*
	Median	Interquartile range	Median	Interquartile range	
Females (n, %)	3570 (54%)		324 (44%)		<0.001
Age (years-old)	39	(28, 51)	58	(47, 69)	<0.001
Duration of symptoms (days)	4	(2, 8)	7	(4, 10)	<0.001
Missing data	1452		323		
Systolic blood pressure (mmHg)	133	(121, 146)	129	(114, 145)	<0.001
Unknown	1091		298		
Diastolic blood pressure (mmHg)	84	(75, 94)	80	(70, 90)	<0.001
Unknown	1103		300		
Heart rate (bpm)	90	(80, 101)	96	(85, 110)	<0.001
Unknown	1223		298		
Temperature (℃)	36.50	(36.00, 36.80)	36.50	(36.00, 36.90)	0.007
Unknown	1214		329		
Respiratory rate (bpm)	18	(16, 20)	24	(20, 28)	<0.001
Unknown	1634		320		
SpO_2 (%)	97.00	(96.00, 98.00)	94.00	(90.00, 96.00)	<0.001
Unknown	1038		280		
Influenza vaccine (yes, %)	1897 (39%)		149 (47%)		0.007
Unknown	1743		421		
Fever (n, %)	2500 (42%)		289 (57%)		<0.001
Unknown	632		236		
Fatigue (n, %)	1628 (27%)		184 (37%)		<0.001
Unknown	632		236		
Sneezing (n, %)	496 (8.3%)		20 (4.0%)		<0.001
Unknown	630		236		
Dry cough (n, %)	2702 (45%)		262 (52%)		0.004
Unknown	631		236		
Productive cough (n, %)	720 (12%)		75 (15%)		0.065
Unknown	632		236		
Running nose (n, %)	1304 (22%)		45 (8.9%)		<0.001
Unknown	634		236		
Sore throat (n, %)	1428 (24%)		38 (7.6%)		<0.001
Unknown	634		237		
Diarrhoea (n, %)	851 (14%)		88 (17%)		0.051
Unknown	632		236		
Breathing difficulty (n, %)	1955 (33%)		302 (60%)		<0.001
Unknown	632		237		
Anorexia (n, %)	614 (10%)		94 (19%)		<0.001
Unknown	631		236		

Headache (n, %)	2219 (37%)	93 (18%)	<0.001
Unknown	632	236	
Myalgia (n, %)	1845 (31%)	138 (27%)	0.10
Unknown	630	237	
Nausea/vomiting (n, %)	735 (12%)	91 (18%)	<0.001
Unknown	629	239	
Wheezing (n, %)	136 (2.3%)	11 (2.2%)	0.9
Unknown	631	236	
Thoracic pain (n, %)	1059 (18%)	67 (13%)	0.012
Unknown	631	237	
Abdominal pain (n, %)	320 (5.4%)	34 (6.7%)	0.2
Unknown	630	236	
Anosmia (n, %)	1133 (19%)	80 (16%)	0.084
Unknown	633	236	
Dysgeusia (n, %)	1127 (19%)	80 (16%)	0.10
Unknown	632	237	
Chills (n, %)	672 (11%)	46 (9.1%)	0.14
Unknown	630	236	
Hypertension (n, %)	1227 (21%)	232 (46%)	<0.001
Unknown	621	236	
Cardiac disease (n, %)	227 (3.8%)	68 (14%)	<0.001
Unknown	617	237	
Diabetes mellitus (n, %)	469 (7.8%)	136 (27%)	<0.001
Unknown	619	235	
Cerebrovascular disease (n, %)	42 (0.7%)	17 (3.4%)	<0.001
Unknown	620	235	
Chronic kidney disease (n, %)	162 (2.7%)	66 (13%)	<0.001
Unknown	621	235	
Immunosuppression (n, %)	230 (3.8%)	55 (11%)	<0.001
Unknown	617	236	
COPD (n, %)	95 (1.6%)	13 (2.6%)	0.10
Unknown	621	235	
Asthma (n, %)	355 (5.9%)	19 (3.8%)	0.044
Unknown	620	235	
Tuberculosis (n, %)	39 (0.7%)	6 (1.2%)	0.2
Unknown	618	235	
Other respiratory diseases (n, %)	87 (1.5%)	12 (2.4%)	0.10
Unknown	617	235	
Neoplasia (n, %)	89 (1.5%)	30 (6.0%)	<0.001
Unknown	622	236	
Solid-organ transplant (n, %)	145 (2.4%)	63 (12%)	<0.001
Unknown	619	235	

Obesity (n, %)	305 (5.1%)	56 (11%)	<0.001
Unknown	617	236	
Smoking (n, %)	621 (10%)	45 (8.9%)	0.3
Unknown	616	235	
Pregnancy (n, %)	79 (1.3%)	3 (0.6%)	0.2
Unknown	618	235	
Previous hospitalization (n, %)	37 (0.6%)	23 (4.5%)	<0.001
Unknown	623	233	

SpO_2: oxyhemoglobin saturation by pulse oximetry; COPD: Chronic obstructive pulmonary disease.

Table S2. Predictors and missing values numbers used in predicting hospitalization in patients with respiratory symptoms during the COVID-19 pandemic**.**

Variable	N (not missing)	N (missing)	Missing (%)
Sex	7336	0	0.0
Age	7336	0	0.0
Symptom Duration	5561	1775	24.2
Date symptoms	5578	1758	24.0
Systolic pressure	5947	1389	18.9
Diastolic pressure	5933	1403	19.1
Heart Rate	5815	1521	20.7
Temperature	5793	1543	21.0
Respiratory frequency	5382	1954	26.6
SpO_2	6018	1318	18.0
Influenza Vaccine	5172	2164	29.5
Fever	6468	868	11.8
Fatigue	6468	868	11.8
Cough	6470	866	11.8
Dry cough	6469	867	11.8
Phlegm cough	6468	868	11.8
Running nose	6466	870	11.9
Sore throat	6465	871	11.9
Diarrhea	6468	868	11.8
Dyspnea	6467	869	11.8
Anorexia	6469	867	11.8
Headache	6468	868	11.8
Myalgia	6469	867	11.8
Nausea and vomiting	6468	868	11.8
Chest wheezing	6469	867	11.8
Chest pain	6468	868	11.8
Abdominal pain	6470	866	11.8
Anosmia	6467	869	11.8
Dysgeusia	6467	869	11.8
Chills	6470	866	11.8
Hypertension	6479	857	11.7
Heart disease	6482	854	11.6
Diabetes mellitus	6482	854	11.6
Cerebrovascular disease	6481	855	11.7
Chronic kidney disease	6480	856	11.7
Immunosuppression	6483	853	11.6
COPD	6480	856	11.7
Asthma	6481	855	11.7

Tuberculosis	6483	853	11.6
Respiratory disease	6484	852	11.6
Neoplasia	6478	858	11.7
Transplant	6482	854	11.6
Obesity	6483	853	11.6
Smoking	6485	851	11.6
Pregnant	6483	853	11.6
Prior hospitalization	6480	856	11.7
Hospitalization	7336	0	0.0
Death	7336	0	0.0

SpO_2: oxyhemoglobin saturation by pulse oximetry; COPD: Chronic obstructive pulmonary disease.
